# Research progress on m6A demethylase FTO and its role in gynecological tumors

**DOI:** 10.3389/fonc.2024.1413505

**Published:** 2024-08-08

**Authors:** SiYuan Wang, Qin Liu

**Affiliations:** ^1^ Jiangsu University School of Medicine, Jiangsu University School, Zhenjiang, Jiangsu, China; ^2^ Gynecology, KunShan Affiliated Hospital of Jiangsu University, Jiangsu University, Suzhou, Jiangsu, China

**Keywords:** FTO, M6A, gynecological tumors, therapeutic target, epigenetics

## Abstract

Recent advances in genomic research have increasingly focused on the fat mass- and obesity-associated (FTO) gene due to its notable correlation with obesity. Initially explored for its contribution to increased body weight, FTO was later discovered to function as an m6A demethylase. This pivotal role enhances our understanding of its broader implications across various pathologies. Epigenetic modifications, such as m6A, have been implicated in gynecological cancers, including ovarian, endometrial, and cervical malignancies. However, the precise mechanisms by which FTO influences the development of gynecological cancers remain largely unknown. This analysis underscores the growing relevance of investigations into the FTO gene in elucidating the mechanisms underlying gynecological cancers and exploring potential therapeutic avenues.

## 1 Introduction

The initial focus on the FTO gene was due to its link with obesity, and for an extended period, the impact of obesity on various diseases has been a prominent research topic. Recently, the FTO has emerged as a pivotal subject of study (1–3). A pivotal advancement in the field of epitranscriptomics was achieved with the characterization of FTO as the inaugural m6A demethylase, capable of facilitating dynamic and reversible epigenetic modifications. This discovery has opened new avenues for understanding how genetic information can be regulated post-transcriptionally in a reversible manner. FTO has been implicated in the invasion, migration, and proliferation of tumors across several cancers and is influenced by m6A levels. Research has shown that FTO is involved in critical processes, such as malignant tumor development, immunosuppression, glycolysis, drug resistance, angiogenesis, epithelial-mesenchymal transition, metastasis, and cancer cell proliferation (4–7). Given its significant role in these diverse processes, FTO is a promising target for the detection and management of gynecological tumors including ovarian, endometrial, and cervical cancers.

## 2 Crystal structure of FTO

**Figure 1:**
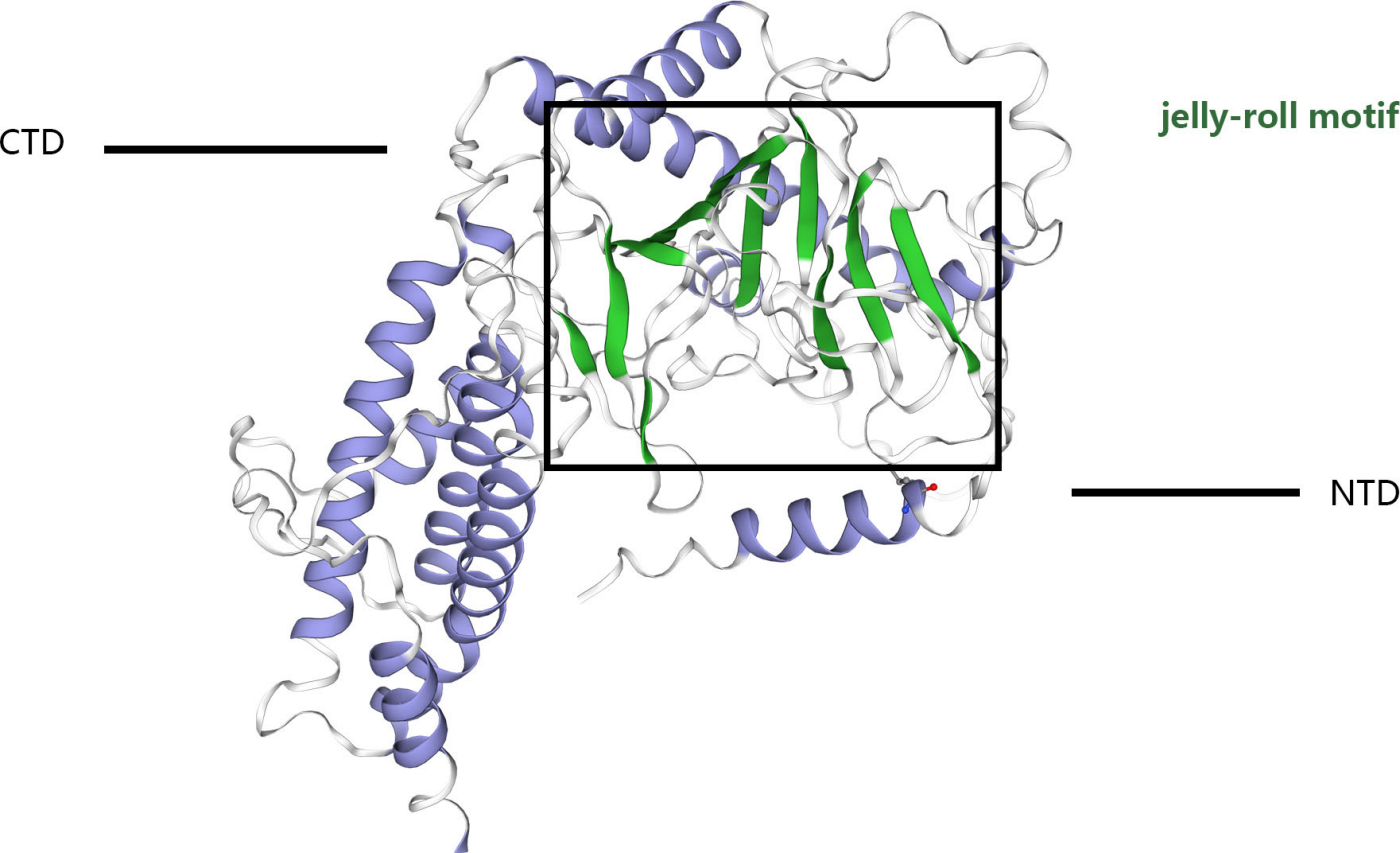
The crystal structure of the FTO protein comprises a catalytic N-terminal domain (NTD) and a C-terminal domain (CTD). The NTD contains a double-stranded b-helix fold termed the jelly-roll motif. The black box shows the jelly-roll motif.

The structural blueprint of the FTO enzyme is partitioned into two distinct domains: a CTD, and an NTD, which orchestrates the catalytic processes (8–12), (**Figure 1**). Gerken et al. (13) have documented that FTO acts as a demethylase that is reliant on both Fe²^+^ and 2-OG, situating it within the broader category of dioxygenase enzymes. A notable feature of FTO is its incorporation of the jelly roll motif, a hallmark of the AlkB family, defined by a double-stranded β-helical structure that maintains high conservation across species. Unique to FTO within its protein family, an additional structural element, the NRL loop, is integrated within its framework. This loop strategically encases one flank of the jelly roll motif, a design that crucially enhances the protein’s capacity for engaging with unmethylated double-stranded DNA actively (14). Moreover, the C-terminal domain is comprised of three α-helical segments, labeled α7, α8, and α10. These segments collectively form a triple-helix bundle that not only fosters substantial interactions with the N-terminal domain but also significantly influences the enzymatic dynamics and overall stability of FTO, thus offering potential insights into its functional versatility and regulation. FTO shows a high affinity for 3-methyl thymidine (3-meT) in single-stranded DNA and RNA and for 3-methyl uracil (3-meU) in single-stranded RNA due to the interaction between the CTD and these molecules (13–15). This structural foundation underpins FTO’s strong affinity of FTO for RNA and DNA. Importantly, FTO proteins exhibit a greater affinity for N6-methyladenosine (m6A) in messenger RNAs (mRNAs), enabling efficient demethylation (5).

## 3 Association of FTO with demethylation

Recent research has underscored the vital influence of epigenetic modifications on the management of physiological functions and the evolution of various diseases. Among these, RNA modifications stand out as a primary means by which RNA governs molecular functions and ensures RNA stability. To this point, m6A is the most common internal RNA modification in mammalian mRNAs (16). Among these, m6A was first detected in poly(A) RNA in the year 1974 and has since been established as the most frequently occurring epigenetic modification within the mRNAs of eukaryotic organisms. Initially, it was considered a widespread modification capable of selectively affecting gene expression (17). The methylation of adenine to m6A in messenger RNA is orchestrated by a methyltransferase enzyme that specifically methylates the nitrogen atom located at position six of the adenine base. This epigenetic alteration is predominantly localized near the mRNA’s stop codon and within the 3′- UTR, which enhances cap-independent translation mechanisms adjacent to the 5′-UTR (18–20). Compelling evidence is accumulating which indicates that the m6A modification plays a comprehensive role in modulating various aspects of mRNA metabolism. This modulation is crucial for maintaining cellular homeostasis and has been implicated in the development of diverse pathological conditions, including various forms of cancer (21). The discovery of methyltransferases (writers), demethylases (erasers), and reader proteins (readers) highlighted that m6A RNA methylation is a dynamically reversible biological process, with demethylases enabling the conversion of m6A to A (22). M6A RNA modifies a variety of non-coding RNAs (ncRNAs) (23). It influences mRNA transcription, splicing, translation, localization and metabolism in the nucleus and further affects gene expression, leading to a range of pathophysiological processes (24–26). The realization of these effects requires the synergistic action of m6 functional enzymes to achieve a dynamic balance of the intracellular regulatory network. For example, m6A modifications are often enriched in the intronic region of pre-mRNAs in mRNA splicing, and its regulators are mainly located in the cytosolic speckles (27, 28). The modifications regulating mRNA splicing are indispensable to the action of m6A transferase (such as METTL3) and m6A eraser (such as FTO) (29–31). For example, multiple translation mechanisms are closely associated with the m6A reader YTHDF1 and the methyltransferase METTL3 during mRNA translation (26, 32, 33). Most of the residues of m6A are located in the exon closest to the 3’-most (last) exons, while they and more than 40% of all m6A in mRNAs are present in 3’-UTRs (34). FTO regulates the expression of the last exon, and FTO affects the expression and processing of the 3-terminus of mRNAs. This implicates FTO in the selective regulation of the poly(a) site and in the control of 3’-UTR length. Similarly, this study found that METTL3 and FTO have opposing roles in regulating the expression of intronic and terminal exons (35). FTO acts on m6A by initially oxidizing it to N6-hydroxymethyl adenosine (hm6A), an intermediate in the demethylation process. Subsequently, FTO oxidizes hm6A to N6-formyl adenosine and ultimately to adenosine (A) (36). The function of the additional loop surrounding one side of the conserved jelly roll motif may be associated with FTO’s target specificity of FTO in this mechanism (14). Further substantiation of m6A’s role in regulating RNA processing has been demonstrated through its simultaneous localization with components of both the FTO and the m6A methylation complexes, as well as with splicing proteins within the confines of nuclear speckles. These speckles are integral to the orchestration of mRNA processing factors, facilitating the assembly and function of the molecular machinery required for RNA modification and splicing (5, 37). Mauer et al. (22) proposed (38) that FTO’s demethylation activity on m6Am is approximately 100 times higher than that on m6A, suggesting preferential demethylation of m6Am by FTO. Additional studies suggested that the preference of the FTO substrate for demethylation is influenced by its cellular localization. In the cytoplasm, FTO targets m6Am in the cap region and m6A in mRNA, whereas in the nucleus, its substrates include m6A in snRNA and mRNA (39). He et al. demonstrated that the distribution of FTO exhibits notable variation across different cell lines, specifically between the cytoplasm and nucleus. They found that within the cytoplasm, FTO primarily interacts with m6Am, whereas in the nucleus, it predominantly focuses on m6A, which acts as the principal substrate for polyadenylated RNA (39). FTO’s high affinity for m6Am is attributed to the unique structure of the cap region, which effectively attracts FTO despite the structural differences in the five-carbon sugar rings of m6A and m6Am. Within identical RNA sequences, FTO’s catalytic activity of FTO towards both m6A and m6Am remains consistent (40). According to recent reports, METTL3 can regulate chromatin-associated regulatory RNA (car RNA) through m6A methylation, thereby affecting chromatin status and transcription in FCT embryonic stem cells, whereas FTO can alter the rate of LINE1 RNA degradation and expression, thereby regulating chromatin status in mammalian tissues and during development (41). FTO has been identified as a significant factor in a broad spectrum of human health issues, including obesity, diabetes mellitus, cardiovascular disorders, neurological anomalies, and various oncological conditions. Specifically within the realm of cancer research, FTO is recognized for its critical role in facilitating tumor progression, promoting invasive behavior, and enabling cellular migration. These oncogenic processes are largely driven by its regulation of m6A RNA modifications, which are pivotal in controlling gene expression related to cancer dynamics (4–7). Current research efforts are exploring the role of FTO in the development of gynecological cancers. This investigation aims to uncover how FTO contributes to the dynamics of tumor growth and spread within these specific malignancies (**Table 1**).

**Table 1:** FTO affects 3 main gynecological malignancies.

	Cancer	Function	Target	References
FTO	Cervical Cancer	Proliferation	MY、E2F1	(42)
			BMP4/Hippo/YAP1/TAZ	(43)
			HOXCB-AS/FZD6	(44)
		Glycolysis	HK2	(45)
			GSK3β	(45)
		Immunotherapy	PD-L1	(46)
		Radiotherapy Resistance	ERCC1	(47)
	Ovarian Cancer	Cell proliferation and Apoptosis	PI3K/AKT	(48)
			PCNA,Bax, Bcl 2, caspase-3, and caspase-9	(48)
		EMT	SNAI1	(49)
		Stem cell Renewal	PDE1C, PDE4B	(50)
		Radiotherapy and Chemotherapy Sensitivity	FZD10	(51)
			RP5–991G20.1/hsa-miR-1976/MEIS1	(52)
			NNMT/γ-H2AX	(53)
	Endometrial Cancer	Proliferation and Invasion	PI3K/AKT,MPAK	(54)
			HOXB13	(55)

## 4 Biological functions and underlying mechanisms of FTO in gynecological tumors

### 4.1 FTO in cervical cancer

CC stands as the fourth most widespread form of cancer among women globally and constitutes a significant concern within the realm of gynecological oncology (40, 56). An escalating trend in the incidence of cervical cancer highlights a significant peril to the health and survival of women worldwide (57). The predominant therapeutic strategies for combating this disease include a regimen of chemotherapy, surgical interventions, radiotherapy, and their integrated applications (58). The overall prognosis for numerous cervical cancer patients continues to be poor, a situation exacerbated by obscure pathophysiological mechanisms, a high tendency for metastasis and recurrence, limited efficacy of radiation treatments, and an escalation in chemoresistance as the cancer advances (59). Recent studies have increasingly pointed to FTO’s involvement in cervical cancer, suggesting its role in influencing the disease’s progression, as documented in several pieces of research (**Table 2**).

**Table 2:** FTO is associated with multiple genes affecting proliferation, migration, EMT, glycolysis, and sensitivity to radiotherapy in cervical cancer cells.

FTO in cervical cancer				
	correlation	target	function	References
FTO	positive correlation	MYC、E2F1	Proliferation、migration	(42)
	positive correlation	ZEB1	Proliferation、migration	(42)
	positive correlation	BMP4	Proliferation	(43)
	positive correlation	HOXCB-AS/FZD6	invasion, proliferation, and EMT	(44)
	negative correlation	HK2	regulation of glycolysis	(45)
	negative correlation	GSK-3β	regulation of glycolysis	(45)
	positive correlation	β-catenin, ERCC1	Chemo-radiotherapy resistance	(47)

#### 4.1.1 Involvement in cervical cancer cell proliferation

FTO is strongly correlated with cervical cancer progression and is overexpressed in human cervical cancer tissues. In their comprehensive analysis, Wang et al. meticulously examined a substantial cohort of 286 individuals diagnosed with cervical cancer. Their findings highlighted a significant diminution in the levels of m6A methylation across the tumor tissues, suggesting an altered epigenetic landscape in the affected cells (60). Investigations have revealed that the m6A methylation status correlates significantly with crucial clinical outcomes such as patient relapse rates, overall survival rates, and intervals of disease-free survival. Additionally, enhanced expression of the FTO gene within the SiHa cervical cancer cell line has been shown to lead to a reduction in m6A methylation. This decrease in methylation consequently facilitates a notable acceleration in the proliferation of the cervical cancer cells, indicating a potential pathway for targeted therapeutic intervention (5). Subsequent investigations (42) as reported by Cancer Cell International, found that patients with advanced stages of cervical cancer (stages III and IV) exhibited significantly higher expression of FTO than those in the early stages (stages I and II) or those with a normal cervix. This suggests that elevated FTO expression may contribute to the progression of cervical cancer.

The regulatory mechanisms of FTO within the context of cervical cancer have emerged as a crucial area of investigation. Research spearheaded by Wang et al. has elucidated that FTO directly influences the dynamics of cervical cancer cell migration and proliferation through its interactions with key molecular players such as E2F1 and Myc mRNA (42). Additionally, this enzyme plays a critical role in the m6A methylation of ZEB1. Notably, the malignancy-associated behaviors of cervical cancer cells undergoing FTO knockdown are significantly modified by the compensatory overexpression of ZEB1, which mitigates these effects (61).

FTO’s influence on BMP4 regulation indicates a profound indirect contribution to the progression of cervical cancer, observable in both controlled laboratory settings and clinical scenarios. Bone morphogenetic proteins (BMPs), crucial components of the Transforming Growth Factor-β (TGF-β) superfamily, are primarily acidic, hydrophobic glycoproteins sourced from bone matrices. These proteins are pivotal in initiating the development of bone, cartilage, and other tissues, and they play a central role in numerous biological processes (62). BMPs exert a regulatory effect on various cellular functions, including the self-renewal of embryonic stem cells, and their proliferation and growth dynamics. Additionally, the work of Huang et al. has demonstrated that BMP4 treatments not only encourage the growth of cervical cancer cells *in vitro* but also effectively inhibit the Hippo/YAP1/TAZ signaling pathway by reducing FTO levels. This suppression illustrates the potential of FTO as an oncogenic facilitator, suggesting its significance in the molecular pathology of cervical cancer (43).

Furthermore, FTO reduces the m6A level of *Homo sapiens* homeobox C13 antisense RNA (HOXC13-AS) to enhance its stability. Once stabilized by FTO, HOXC13-AS was found to elevate FZD6 levels and activate the Wnt/β-catenin signaling pathway, thereby promoting CC invasion, proliferation, and epithelial-mesenchymal transition (EMT) (44).

#### 4.1.2 Involvement in glycolysis in cervical cancer cells

The phenomenon of aerobic glycolysis stands as a significant characteristic of cancer metabolism and serves as an innovative therapeutic target in the treatment of tumors. This metabolic process is intricately regulated by hexokinase (HK), a vital enzyme in the glycolytic pathway, which is responsible for converting glucose into glucose-6-phosphate, a critical step in cellular energy production (63). It has been well-documented in scientific literature that HK2, a specific isoform of hexokinase, is substantially overexpressed in cervical cancer tissues. This overexpression correlates strongly with increased tumor growth, enhanced capacity for metastatic invasion, and a marked resistance to conventional radiation therapy (64–66). The influence of FTO on cancer metabolism is notable, as it affects the expression levels of HK2 both at the mRNA and protein levels. Overexpression of FTO has been observed to curb the overactive expression of HK2, leading to a modulation of the enzyme’s activity. Moreover, the accumulation of HK2 pre-mRNA in the nucleus, a result of increased FTO activity, impedes the proper maturation and translation of HK2 mRNA, affecting the efficiency of this glycolytic enzyme (45). On the other hand, METTL3, a known m6A methyltransferase, plays a contrasting role by enhancing the stability and subsequent protein synthesis of HK2 mRNA, thereby facilitating an increased glycolytic flux essential for cancer cell proliferation and survival (67). This may allow researchers to better understand the mutual constraints between these two enzymes. The relationship between METTL3 and HK2 has to be studied in more and more detail. It is known that METTL3 targets the 3’-untranslated region (3’-UTR) of HK2 mRNA in cervical cancer cells. Additionally, METTL3 recruits YTHDF1 (an m6A-reading protein) to enhance HK2 stability (67). Furthermore, FTO and ALKBH5 regulate HK2 in an m6A-dependent manner and regulate colorectal cancer cell proliferation through the HK2-mediated FOXO pathway (68). However, there is still a lack of studies explaining how FTO acts on HK2 and what pathway FTO regulates cervical cancer cell proliferation through HK2-mediated. Upregulation of another glycolysis-associated enzyme (glycogen synthase kinase-3β [GSK-3β]) can result in enhanced FTO ubiquitination and reduced FTO protein expression levels. This process may be driven by the human papillomavirus-encoded oncoproteins E6 and E7, which regulate the transcription of GSK-3β through elements located 85 and 250 bp upstream of the promoter’s transcription seven-point (69). Glycolysis, a crucial metabolic pathway in cancer cells, affects several aspects of their malignancy, including cellular proliferation, metastasis, and the formation of new blood vessels. It is therefore imperative to conduct further investigations to unravel the precise mechanisms by which FTO impacts glycolysis in the context of cervical cancer and to examine the interactions between FTO and various regulators of glucose metabolism.

#### 4.1.3 FTO and immunotherapy for cervical cancer

The tumor microenvironment comprises myeloid cells, lymphocytes, fibroblasts, and mesenchymal stem cells. Studies have shown that T cell immunoreceptors with IG and ITIM domains, such as PDL-1 and PD-1, are expressed on the surface of activated T-cells and NK cells within this environment. The abnormal expression of these proteins may facilitate immune escape, potentially contributing to tumor development and progression.

Utilizing the comprehensive datasets from the TCGA and GTEx databases, Ji et al. conducted an extensive analysis by collecting RNA-seq transcriptomic data along with clinical information pertaining to patients diagnosed with cervical cancer. Their study utilized cluster analysis techniques to systematically evaluate several key factors influencing cervical cancer progression. These included PD-L1 expression, immunological scores, the extent of immune cell infiltration, the characteristics of the tumor microenvironment (TME), and other critical pathways associated with cervical cancer. Remarkably, the analysis revealed that PD-L1 expression was significantly elevated in cervical cancer tissues in comparison to the adjacent normal tissues (p < 0.001). The study also identified a robust inverse correlation between the expression of FTO and PD-L1. Additionally, other m6A-related enzymes, such as RBM15B, ALKBH5, ZC3H13, METTL3, YTHDF3, and YTHDF1, were found to exhibit negative correlations with PD-L1 expression (p < 0.01). These findings highlight the significant role that m6A-modifying enzymes, including FTO, may play as potential targets for developing targeted immunotherapeutic strategies in the management of cervical cancer. However, investigations into the relationship between m6A modifications, TME, and cervical cancer are still preliminary, necessitating further research to elucidate the precise mechanisms (46). Therefore, FTO is expected to serve as a novel biomarker for predicting the efficacy of immunotherapy.

#### 4.1.4 Radiotherapy resistance in cervical cancer

Beta-catenin, a marker of EMT, is associated with resistance to anticancer therapies. Zhou et al. found that FTO expression was significantly elevated in the tumor tissues of patients with uterine cervical squamous cell carcinoma who exhibited resistance to radiotherapy. Treatment with the FTO inhibitor meclofenamic acid enhanced radiation sensitivity of cervical squamous cell carcinoma cells. This effect may be attributed to FTO’s influence on reducing the expression of ERCC1 and decreasing m6A levels in the transcriptome of β-catenin mRNA. Furthermore, it was found that patients with CSCC exhibiting high levels of both β-catenin and FTO faced a poorer prognosis (P = 0.041) compared to those with elevated FTO expression alone (47). Therefore, targeting FTO could enhance the efficacy of radiotherapy in the treatment of cervical cancer.

### 4.2 FTO in ovarian cancer

Ovarian cancer, known as OC, stands as a frequently encountered malignancy impacting the female reproductive tract. Notably, epithelial ovarian cancer represents the most prevalent form of this disease, encompassing approximately 90 percent of all ovarian cancer cases diagnosed globally (70). It is unfortunate that a relatively small percentage of patients with ovarian cancer receive a diagnosis during the early phases of the disease. The conventional approach to managing this malignancy involves initial surgical removal of the tumor, which is typically followed by a chemotherapy regimen that primarily uses a combination of paclitaxel and platinum-based compounds. This combination has been shown to achieve clinical remission in approximately 60 to 80 percent of treated cases. Despite these rates of remission, the treatment is associated with a significant risk of both metastasis and recurrence. As a result, ovarian cancer remains the most lethal malignancy within the realm of gynecological cancers, underscoring the urgent need for more effective management strategies (71).

#### 4.2.1 Involvement in the regulation of ovarian cancer cell proliferation and apoptosis

The role and impact of FTO within ovarian cancer contexts present ongoing subjects of debate among researchers (**Table 3**). Research indicates a significant downregulation of FTO expression in ovarian cancer, as highlighted by several studies (50, 51, 72). The PI3K/AKT pathway, a well-established intracellular signaling system that is active during the cell cycle, involves upstream and downstream molecules that regulate its activation. This pathway is closely associated with cellular physiology (73). In their comprehensive research, Zhao et al. (48) documented a pronounced upregulation of FTO gene expression in tissue samples derived from human ovarian tumors, illustrating its significant involvement in promoting phosphorylation processes, particularly of the RAC-alpha serine/threonine-protein kinase (AKT) in ovarian cancer cells. The study further highlighted that an increase in FTO expression significantly augments the levels of Proliferating Cell Nuclear Antigen (PCNA), a biomarker widely recognized for its association with cellular proliferation dynamics. Additionally, Zhao et al. observed that a surge in FTO expression is linked to substantial reductions in apoptotic activity within ovarian cancer cells, as evidenced by decreased counts of apoptotic cells and distinct alterations in the expression patterns of critical apoptotic markers, including Bax, Bcl2, caspase-3, and caspase-9. These changes underscore the potential of FTO as a key modulator in the survival mechanisms of ovarian cancer cells (48). In contrast to FTO, METTL3, which acts as an m6A methylase, also affects the expression of apoptosis-associated factors such as Bax and Bcl2, but in an opposite manner (74). At present, there remains a lack of consensus within the medical research community concerning the specific influence of FTO on the diverse pathological subtypes of ovarian cancer.

**Table 3:** Expression of FTO in ovarian cancer varies across studies in different.

	Author	Trend	Pathological type	Target	Function	References
FTO	Huang, H., et al.	↓	HGSOC	PDE1C\ PDE4B	FTO expression is downregulated *in vivo* and *in vitro*. (Tumorigenicity and Stemness Characteristics)	(50)
	Cai, Y., et al.,	↓			Database indicated that FTO expression is downregulated in ovarian cancer. (Overall Survival (OS) and Progression-Free Survival (PFS))	(42)
	Sun, M., et al.,	↓	EOC	SNAI1 \IGF2BP2	FTO expression is negatively correlated with the FIGO stage in patients with epithelial ovarian cancer. (Migration; Proliferation; EMT)	(49)
	Zhao. L, et al.	↑		Bcl2\Bax\caspase-9\caspase-3\ATG-5\AKT	FTO expression is elevated *in vitro*. (Proliferation,; Apoptosis; Autophagy)	(48)

↑, FTO expression is upregulated.

↓, FTO expression is downregulated.

#### 4.2.2 Involvement in the regulation of EMT in ovarian cancer cells

EMT is a pivotal biological process in tumor invasion and migration and is characterized by epithelial cells losing their polarity and acquiring mesenchymal traits (75). EMT plays a significant role in the dynamics of tumor invasion and migration.

In epithelial ovarian cancer (EOC), increased m6A levels were associated with decreased FTO expression. Furthermore, research indicates a significant correlation between lower levels of FTO expression and the presence of more advanced FIGO stages in patients suffering from EOC. On a molecular level, FTO exerts its influence by restricting the m6A demethylation of SNAI1 mRNA. This restriction effectively leads to a marked decrease in the stability of SNAI1 mRNA, culminating in lowered SNAI1 expression. These biochemical events play a crucial role in reducing the EMT, which in turn significantly restricts the developmental and metastatic potential of EOC cells, acting as a barrier to tumor aggressiveness (49).

#### 4.2.3 Involvement in ovarian cancer stem cell renewal

Cancer stem cells (CSC) are distinguished by their capacity for self-renewal, growth, differentiation, and tumor generation. These factors have been linked to ovarian cancer metastasis and recurrence after chemotherapy (76, 77).

FTO significantly influences the progression of ovarian cancer by impairing the self-renewal processes of ovarian stem cells and interfering with the cAMP signaling cascade through its function as a demethylation enzyme. Specifically, elevated levels of FTO enhance the efficacy of the 3′,5′-cAMP second-messenger signaling pathway. This enhancement subsequently leads to increased mRNA stability for the phosphodiesterase genes PDE1C and PDE4B, while concurrently reducing the m6A modifications found within the 3′- UTRs. These molecular interactions contribute to a marked reduction in the stem cell-like properties of ovarian cancer cells, which in turn plays a crucial role in slowing the overall growth and proliferation of the tumor (50).

#### 4.2.4 Participation in ovarian cancer radiotherapy and chemotherapy sensitivity 

In epithelial ovarian cancer cells harboring a BRCA1/2 gene mutation, downregulation of FTO can enhance the stability of FZD10 mRNA via m6A modification, leading to elevated expression of FZD10 protein. This process upregulates the Wnt/β-catenin signaling pathway, potentially promoting resistance to PARP inhibitors.

Furthermore, recent research indicates that FTO can significantly curtail both the proliferation and chemoresistance of OC cells by modulating the RP5–991G20.1/hsa-miR-1976/MEIS1 signaling pathway (52). This study highlights the pivotal role of FTO in reducing the aggressive growth and enhancing the chemosensitivity of OC cells, establishing it as a key player in the battle against tumor progression and therapeutic resistance. The inhibition of FTO expression notably led to increased proliferation rates and heightened resistance to chemotherapeutic agents like CDDP and PPARi in A2780 ovarian cancer cells, alongside a decrease in apoptotic activity. These observations suggest that strategic manipulation of these signaling pathways could offer a novel method to substantially improve the responsiveness of OC to immunotherapy and combat chemotherapy resistance, providing new avenues for treatment optimization in ovarian cancer (52). According to a separate study, cells overexpressing FTO, after exposure to Pt, exhibited elevated levels of γ-H2AX foci, increased quantification of DNA double-strand breaks, and enhanced apoptosis compared to control cells. FTO overexpression stimulates the demethylation of nicotinamide N-methyltransferase (NNMT), thereby augmenting NNMT expression. Upon treatment with NNMT inhibitors or NNMT knockdown, cells overexpressing FTO regained sensitivity to Pt. NNMT is a potential novel target of FTO associated with Pt response (53). This is concerning because the existing data only suggest that FTO expression influences the sensitivity of patients with ovarian cancer to chemotherapy, and further evidence from randomized controlled trials offering a higher level of evidence is necessary to substantiate this conclusion (**Table 4**).

**Table 4:** FTO affects the resistance of ovarian cancer to chemotherapy.

	Cell lines	Infection	References
FTO in ovarian cancer	FZD-WNT\β-catenin	PPARi resistance	(51)
	NNMT\Pt-DNA adducts\γ-H2AX\caspase-7	Pt resistance	(53)
	RP 5 - 991 G20. 1/hsa-miR-1976/MEIS 1	cDDP and PPARi Resistance	(52)

### 4.3 FTO in endometrial cancer

Endometrial cancer (EC) is the malignant growth of the uterine epithelium and is categorized into two types: type I, associated with non-antagonistic estrogen stimulation, and type II, which is estrogen-independent. Over the past two decades, EC has emerged as the predominant cancer affecting the female reproductive system in Europe and the US (78). The annual incidence of endometrial cancer has been increasing worldwide. A substantial number of EC cases in North America and Western Europe are largely attributed to lifestyle factors, with obesity playing a particularly significant role. It is estimated that around 50% of these EC cases are directly linked to excessive body weight and associated lifestyle choices, underscoring the critical impact of obesity on the prevalence of this type of cancer (79, 80).

Patients exhibiting high levels of FTO expression in endometrial cancer tissues tend to have significantly shorter periods of DFS and OS in comparison to those with lower FTO expression. This correlation suggests that elevated FTO levels may be linked to more aggressive disease progression and poorer prognosis in endometrial cancer patients (54).

Increased FTO expression has also been observed in endometrial cancer tissues. Despite comprehensive statistical analyses of clinical data revealing no significant correlation between heightened FTO protein expression and various characteristics of endometrial cancer—including patient age, disease stage, tumor grade, extent of infiltration, and lymph node metastasis—high levels of FTO are nevertheless associated with a poorer prognosis and earlier recurrence in affected patients (54). Furthermore, extensive database analyses have identified a relationship between increased FTO expression and decreased overall survival rates (49). These findings underscore the potential of FTO as a critical prognostic biomarker for endometrial cancer, suggesting that it could be instrumental in predicting disease outcomes and guiding treatment strategies.

In a 2012 study, Zhang et al. revealed that E2 significantly accelerates the proliferation and invasion of endometrial cancer cells by activating the FTO enzyme through the PI3K/AKT and MAPK signaling pathways. This activation triggers the upregulation of key proteins such as Cyclin D1 and MMP2/9, which are vital for promoting cell growth, invasive behavior, and migratory capabilities (81). Building on this, Zhu et al. found that endometrial cancer tissues with elevated levels of estrogen receptor α (ERα) show increased nuclear accumulation of FTO. Their research also demonstrated that estrogen enhances FTO mRNA levels in endometrial cancer cells in a dose-dependent manner, with the most pronounced effects occurring at concentrations of 9–10 mol/L. Furthermore, E2 supports the growth of endometrial cancer cells by activating the mTOR signaling pathway, which is crucial for cell proliferation (54). Additionally, E2-induced FTO further promotes cell proliferation and invasion by activating both the PI3K/AKT and MAPK pathways. This underscores the significant role of these signaling mechanisms in the progression and aggressiveness of endometrial cancer (54).

Delahanty et al. conducted a study to explore the relationship between FTO gene variants and the risk of endometrial cancer. Their findings revealed that certain SNPs in the FTO gene did not show any correlation with BMI. However, these same SNPs were significantly associated with an increased risk of endometrial cancer. This suggests that the FTO gene may influence the development of endometrial cancer through biological pathways that are independent of obesity. These insights reveal a novel aspect of FTO’s role in cancer biology, indicating that its impact on endometrial cancer is not merely a consequence of its association with body weight but involves distinct mechanisms that warrant further investigation (82).

When compared to paraneoplastic or normal endometrial tissues, Liu (83)et al. (71) discovered that m6A methylation was reduced in approximately 70% of endometrial tumors. They also found that altered m6A methylation levels in mRNAs contributed to the development of endometrial cancer. Extensive research has illuminated the crucial role of FTO in modulating the m6A methylation status of HOXB13 mRNA. By reducing m6A methylation on HOXB13 mRNA, FTO prevents the YTHDF2 protein from recognizing and binding to these methylation sites. This action significantly slows down the degradation of HOXB13 mRNA, resulting in elevated levels of HOXB13 protein. The increased presence of HOXB13 protein then triggers the activation of the WNT signaling pathway and its downstream target proteins, thereby greatly enhancing the metastatic and invasive capabilities of endometrial cancer cells (55). Despite these insights, the detailed relationship between variations in the FTO gene and the incidence or progression of endometrial cancer remains poorly understood. To thoroughly elucidate this connection, further research involving larger, more diverse patient populations is necessary. Such studies could provide deeper insights into the mechanistic role of FTO in endometrial cancer and pave the way for the development of novel therapeutic approaches aimed at targeting this pathway.

## 5 Conclusion

FTO markedly influences the development and prevalence of various gynecological diseases of the female reproductive system. An increasing number of studies indicates that m6A demethylase, a pivotal enzyme in the m6A methylation pathway, plays a role in gynecological cancers. However, documentation of its involvement in rare gynecological malignancies such as vulvovaginal cancer, uterine sarcoma, and fallopian tube cancer remains limited. Despite considerable progress in this domain, the mechanism of action and potential side effects of FTO demethylase are not well understood because of its insufficient application in extensive clinical cohorts. Numerous studies have presented conflicting views regarding FTO’s role in the diverse pathological manifestations of ovarian cancer. Investigating FTO’s utility as a biomarker for the diagnosis and prognosis of gynecological tumors, along with its safety, efficacy, specificity, and sensitivity, is imperative. According to various studies, FTO has significant investigative value for a range of *in vitro* and *in vivo* stimuli, including tumorigenesis, radiation responsiveness, and other mechanisms. Such insights may pave the way for novel interventions for treating gynecological malignancies.

## Author contributions

SW: Writing – original draft. QL: Funding acquisition, Project administration, Supervision, Writing – review & editing.
